# The influence of liposomal quercetin on liver damage induced by microwave ablation

**DOI:** 10.1038/s41598-017-13010-1

**Published:** 2017-10-04

**Authors:** Xuhua Duan, Pengfei Chen, Xinwei Han, Jianzhuang Ren, Zhaoyang Wang, Guorui Zhao, Hao Li

**Affiliations:** Department of Radiology, The First Affiliated Hospital, Zhengzhou University, No. 1, East Jian She Road, Zhengzhou, 450052 Henan Province People’s Republic of China

## Abstract

This study aimed to observe whether liposomal quercetin (LQ) can enhance the effect of microwave ablation (MWA) on hepatic parenchyma destruction. Forty-eight rabbits were randomly divided into three groups: LQ group, MWA group and LQ + MWA group. Serum and liver samples were collected. The coagulation volume (CV) of hepatic parenchyma, histopathological changes and liver function were compared. Hepatocyte apoptosis was examined through TUNEL. The expression of heat shock protein 70 (HSP70), hypoxia-inducible factor-1α (HIF-1α) and tumor necrosis factor-α (TNF-α) were analyzed. Compared with MWA group, the CV of coagulation necrosis in liver was significantly increased in LQ + MWA group. TUNEL results showed that the hepaocyte apoptosis was higher in LQ + MWA group than MWA group on 12 h, 24 h and 3 d, respectively. HSP70 and HIF-1α expression in both MWA group and LQ + MWA group were increased at 12 and 24 hours, peaked on day3 and dropped on day7. Compared with MWA group, HSP70 and HIF-1α expression were lower in LQ + MWA group. On the contrary, TNF-α expression was decreased in MWA group and LQ + MWA group compared with LQ group. In conclusion, LQ increased hepatocyte apoptosis and MWA-induced hepatic parenchyma destruction through suppressing HSP70 and HIF-1α expression in liver surrounding ablation zone and increasing TNF-α expression.

## Introduction

Hepatocellular carcinoma (HCC) is one of the most common liver neoplasms worldwide. Due to its heterogeneity in biological behaviors and etiology, the treatment effect and survival rate is still bad^1^. In recent years, the thermal ablation techniques acquired great attention for their efficacy and safety^2–5^.

As one kind of thermal ablation technique, MWA therapy has become a new therapeutic option with the promise of generating higher tissue temperatures in shorter durations, larger coagulation zones and less heat sink effect than radiofrequency ablation (RFA)^6,7^. With the improvement of electrode and microwave generator, MWA has been demonstrated the feasibility and efficacy of treating small HCC measuring < or = 3.0 cm in diameter^5,8^. However, it is difficult in achieving a complete ablation when tumor’s diameter is larger than 3–5 cm^5,9^. To increase the ablation effect of MWA, preinjected NaCl solution, segmental hepatic blood flow (both arterial flow and portal flow) occlusion or transcatheter arterial chemoembolization (TACE) were tested as combination therapies^2,3,10^. These combination therapies increased tumor coagulation volume (CV) by modulating biophysical properties such as perfusion-mediated tissue cooling, which prevent uniform heating of the entire tumor volume to a temperature sufficient for inducing coagulation necrosis^11,12^. However, preinjected 0.9% or 10% NaCl solution do not benefit coagulation volumes in MWA as those in RFA in an *ex vivo* porcine liver^10^. Occlusion of both arterial flow and portal flow using 2 balloon catheters are complex in the real clinical operations^5^ So, identifying new method to completely ablate is necessary for clinical practice.

Studies showed that heat shock protein 70 (HSP70) was observed in residual tumor cells surrounding the ablation zone and had protective effects against apoptosis^13,14^. In addition, another study showed that significant expression of HSP70 was detected in the transition zone surrounding the MWA-induced ablation zone, especially with low power in rat livers^15^. Ke *et al*. reported that high expression of HIF-1α in surrounding ablation zone contributed to residual tumor rapid growth after low temperature of RFA or insufficient RFA^16^. Wan *et al*. found that incomplete RFA treatment promoted HSP70 and HIF-1α expression in the transition area^17^. HSP70 interfered with the signaling pathways and cellular responses to hypoxic stress and influenced the stability of HIF-1α^18^. These studies suggested that HSP70 and HIF-1α may contribute to the incompletion of ablation.

Quercetin (3,3′,4′,5,7- pentahydroxy-flavone) is one of the most abundant flavonoids in fruits and vegetables. Large number of studies showed that it had anticancer and anti-inflammatory effects^19^. Studies showed that liposomal quercetin (LQ) treatment increased apoptosis and improved RFA-induced tumor destruction by suppression of HSP70 production^14,20^. Thermal ablation also effectively increased LQ concentrations in tumor tissues to promote apoptosis^13^. These studies imply that LQ has the potential to increase the efficacy of ablation. In this study, we will observe whether LQ can enhance the effect of microwave ablation (MWA) on hepatic parenchyma destruction and examine the expression of HSP70 and HIF-1α to clarify the possible mechanism.

## Materials and Methods

### Animals and grouping

Forty-eight Japanese white rabbits (2.0–2.5 kg) were used in this study and anesthetized by intravenous injection of 30 mg/kg sodium pentobarbital. Forty-eight rabbits were randomly divided into three groups (n = 16 each group): IV LQ group (0.5 mg/kg), MWA group (40 s, 30 W) and LQ + MWA group (IV LQ (0.5 mg/kg) and MWA (40 s, 30 W) 24 h later). The entire liver was harvested at four time points: 12 h, 24 h, 3d and 7d after treatment. All experiments were approved by the Animal Care Committee of Henan Province and all experiments were performed in accordance with relevant guidelines and regulations.

### LQ preparation

LQ was prepared in the pharmaceutical laboratory of Zhengzhou University and described briefly as follows:^14^ the mixtures of lecithin/cholesterol in 5:1 weight ratio were dissolved in chloroform/diethyl ether (5:3, v/v) and quercetin was dissolved in absolute ethyl alcohol. Then, the mixtures of lecithin/cholesterol/quercetin were dissolved and evaporated to dryness under reduced pressure at 37 °C in a rotary evaporator. The dried lipid film was then rehydrated with 4 ml of deionized water and the preparation was sonicated with a Sonic Dismembrator at a power output of 200 μA for 240 s at 0 °C (work for interval 3 s). The liposomal size was 114 nm ± 12 nm. The zeta-potential was −38.9 ± 3.3 mV. The polydispersity coefficient was 0.356 ± 0.084.

The LQ formulation was tested and stable for 10 days at 4 °C in deionized water.

### MWA

The MWA was performed with a 2450 ± 50 MHz microwave generator (YIGAO, Nanjing, CHINA) and a 14 G monopolar electrode. The epigastriums were shaved before the MWA procedure and the strict sterile technique was used in entire procedure. The left lobe of rabbit’s liver was exposed through a subxiphoid abdominal incision. The 1 cm tip of 14 G monopolar electrode was inserted into the medial left lobe of liver. The power output was set to 30 W and lasted for 40 s for each rabbit of MWA and LQ + MWA groups. In order to prevent needle track bleeding, a thermocoagulation was performed along the needle track when the ablation finished.

### Evaluation of coagulation volume

The entire liver was harvested after the rabbits were sacrificed. The ablation zones were sectioned at 3–5 mm intervals The largest slice was soaked in 2% 2,3,5-triphenyl staining (TTC) at room temperature for 15 min to detect the extent of coagulated zones and the surrounding infarcted zones. As previously reported, viable tissue with mitochondrial enzyme activity can be stained red by incubating thin tissue sections in TTC at room temperature for 15 min while ablation tissue couldn’t be stained^21^, so that the extent of visible coagulation can be measured. Following the replacement of TTC with phosphate-buffered saline (PBS), an image was taken. Based on the maximum long-axis and short-axis, the diameters of the central discolored region were measured by two observers. The coagulation volume (CV) was calculated according to the following formula: CV = (a * b2)/2, where “a” and “b” is the long and short axis of the CV, respectively. After measurement, the largest slice was preserved in 4% paraformaldehyde for pathological analysis.

### Haematoxylin and eosin (HE) staining

The tissue samples were fixed in paraformaldehyde and sectioned at a thickness of 4 μm. According to a standard protocol, H&E staining was performed.

### AST and ALT

Blood samples were collected from auricular vein of rabbits at 12 h, 24 h, 3 d and 7 d after treatment. Serum levels of AST and ALT were measured using standard enzymatic procedures.

### Immunohistochemistry (IHC)

IHC staining was performed by the EnVision nonbiotin horseradish peroxidase detection system (Dako, Glostrup, Denmark). Briefly, slides were heated in citrate buffer (0.01 M, pH 6.0) for 16 min in a microwave oven, and endogenous peroxidase was blocked with methanol containing 3% hydrogen peroxide for 10 min. For immunohistochemical detection of HSP70 and HIF-1α, the specimens were incubated overnight at 4 °C with mouse anti-HSP70 (dilution, 1:50; ABR, Golden, CO) and mouse anti-HIF-1α (dilution, 1:80; ABR, Golden, CO) monoclonal antibodies, respectively. The sections were then incubated with an anti-mouse secondary antibody (Dako) for 30 min at room temperature, and binding reactions were visualized by DAB (3-30-diaminobenzidine tetrahydrochloride) substrate. Nucleus was lightly counterstained with hematoxylin.

Five photographs were taken for each specimen at ×200 magnifications. The integrated optical density (IOD) of HSP70 expression was analyzed through Image-Pro Plus software (version 6.0, Media Cybernetics, Bethesda, MD, USA) HIF-1α staining was imaged at the periablational rim at 40× magnification and analyzed through Micron Imaging Software (Westover Scientific, Inc, Mill Creek, Washington) to determine percent cell positivity^22^.

### Western blot

Tissue samples were taken from the marginal area (0.5 cm) of the ablated tissues as Liu *et al*. reported^23^. After homogenization of liver tissue, total protein was extracted using protein extraction buffer, containing protease inhibitors and sodium orthovanadate (Santa Cruz Biotechnology, Santa Cruz, CA, USA). A pierce BCA protein assay kit (Pierce Biotechmology, Rockford, IL, USA) was used to determine the protein concentration. Protein was electrophoresed on sodium dodecyl sulfate polyacrylamide gels (Bio-Rad Laboratories, Inc., Hercules, CA, USA) and transferred to pure nitrocellulose membranes (Bio-Rad). Membranes were blocked with 5% nonfat dry milk in Tween-20 buffered TBS (13TBS, 0.1% Tween-20). Membranes were incubated with corresponding primary antibodies against HSP70 (Abcam PLC.), HIF-1α (Abcam PLC.) and β-actin (Streegen, Victoria, BC, Canada), followed by horseradish peroxidase–conjugated secondary antibodies (Streegen). An enhanced luminescence Western blotting detection reagent (GE Healthcare) was used for chemiluminescence and photographed by Versa-Doc model 5000 imaging system (Bio-Rad).

### ELISA

Serum TNF-α was examined through ELISA Polyclonal TNF-α goat anti-rabbit antibodies (USCN Life Science, Wuhan, China) was employed.

### Real time polymerase chain reaction (RT-PCR)

Total RNA was extracted from the specimens using the TRIzol reagent (Aidlab, Beijing, CA, China). The first-strand cDNA was synthesized using a reverse transcription kit (GeneCopoeia, USA) PCR reactions were carried out using a SYBR qPCR mix (Toyobo, Japan). After completion of the PCR cycles, a dissociation curve was obtained. Relative expression of HSP70 mRNA was quantified and expressed as 2^−△△CT^. The primers were: HSP70: 5′-AAGCCAGACGACAATCAGGA-3′ and 5′-CATGGCTGCAAGAACCTCTG-3′; HIF-1α: 5′-GCATCTCCGTCTCCTAACC-A-3′ and 5′-ACACGTTAGGGCTTCTTGGA-3′; and β-actin: 5′-TGGCTCT-AACAGTCCGCCTAG-3′ and 5′-AGTGCGACGTGGACATCCG-3′.

### TUNEL

The TUNEL kit (Roche, Mannheim, Germany) was used to detect hepatocyte apoptosis in liver tissues. Five fields of each sample were evaluated. The results were scored semi-quantitatively by averaging the number of TUNEL positive cells per field at magnification ×200.

### Statistical analysis

Statistical analysis was performed using SPSS 13.0 (SPSS, Chicago, IL, USA). The data were expressed as mean ± SD. The level of HSP70, HIF-1α and TNF-α, and the frequency of apoptotic cells were compared between two time points or groups using ANOVA for repeated measures with Tamhane’s T2 method for multiple comparisons. The Mann-Whitney U test was used to compare ALT and AST, HSP70mRNA and HIF-1αmRNA expressions between two groups at different time points. The significance was assured when the p-value was less than 0.05.

### Ethic Approval

All experiments were approved by the Animal Care Committee of Henan Province and all experiments were performed in accordance with relevant guidelines and regulations.

### Effect of different treatments on hepatic parenchyma destruction

**Figure 1:**
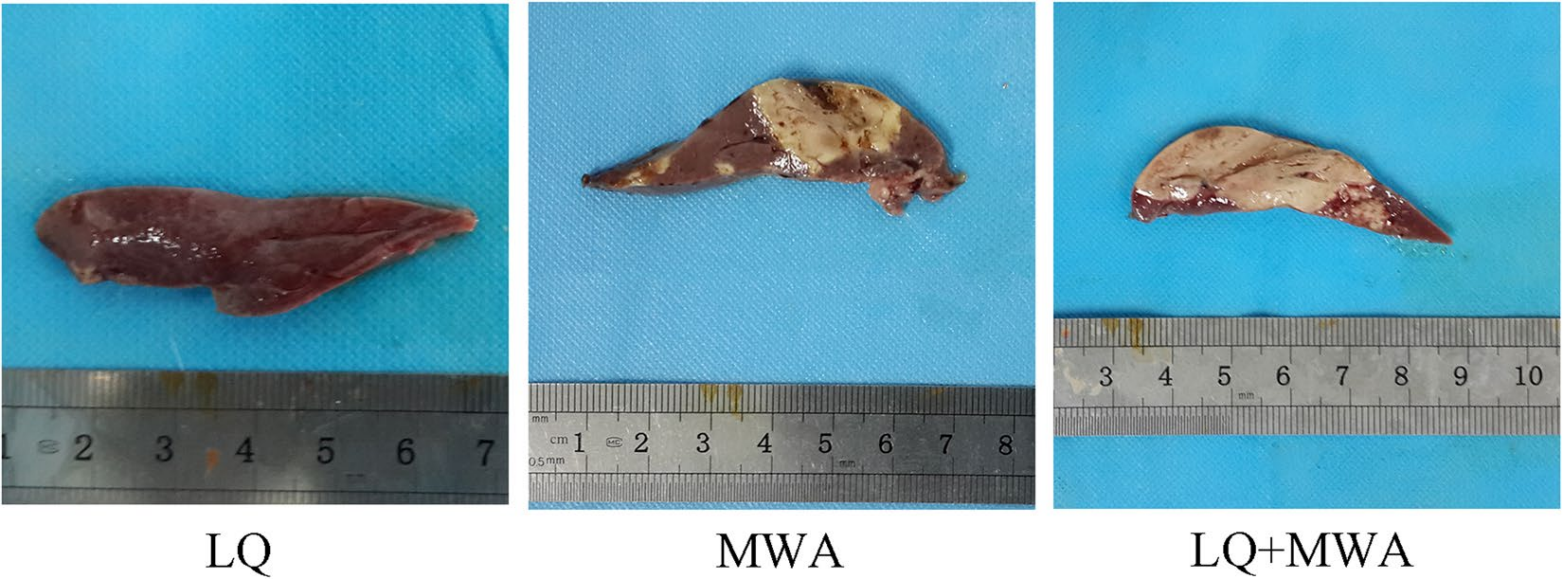
Necrosis zone examination in livers. The coagulation necrosis in hepatic parenchyma was stained with TTC. There was no necrosis zone in LQ group (**A**). The ablated area with irregular margin was showed in the MWA group (**B**). The larger zone with regular margin was shown in the LQ + MWA group (**C**).

The CV of coagulation necrosis in hepatic parenchyma was measured base on the results of TTC staining to reflect the destruction of livers. LQ had no obvious influence on coagulation necrosis (0 mm^3^). LQ + MWA induced more regular ablation margin and greater CV compared with MWA (5414.7 ± 42.14 mm^3^ *vs*. 3002.2 ± 38.84 mm^3^, P < 0.001) (Fig. 1).

**Figure 2:**
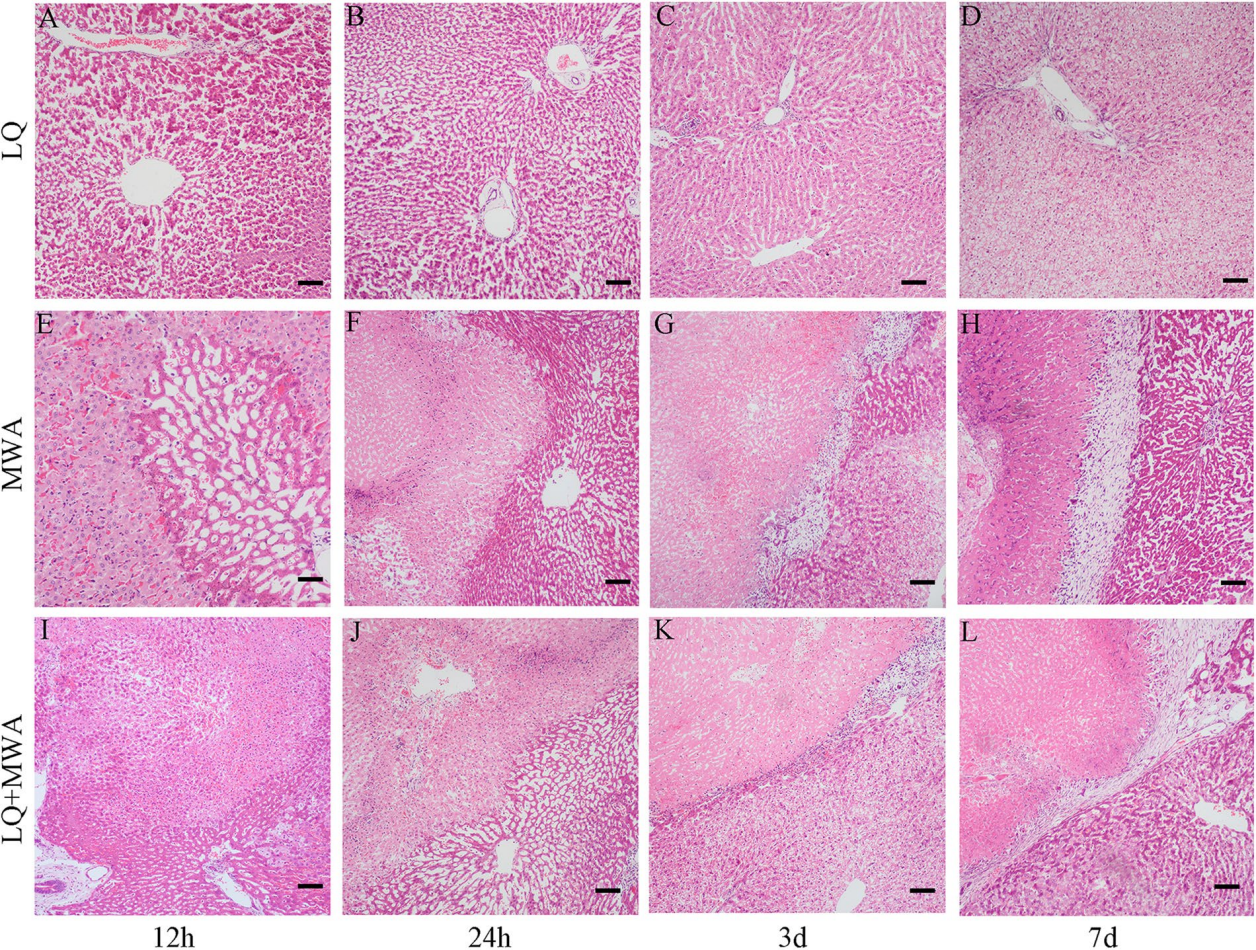
Histopathological changes in livers. HE staining was used to assess the histopathological changes in livers. In LQ group, histopathological changes at different time points (12 h, 24 h, 3 d and 7 d) were shown in (**A**–**D**). (**E**–**H**) Showed that changes of MWA group at 12 h, 24 h, 3 d and 7 d. (**I**–**L**) Showed that changes of LQ + MWA group at 12 h, 24 h, 3 d and 7 d. Scale bar = 100 μm.

Histopathological analysis showed that swollen hepatocytes and necrotic hepatocytes were scattered in the liver on 12 and 24 hour in the LQ group. However, the hepatocytes became normal in morphology on day 3 and 7. The typical coagulation necrosis zone with ‘ghost’ cells, hemorrhagic zone and normal hepatocytes were shown in MWA and LQ + MWA groups on 12 and 24 hours. More eosinophilic ‘ghost’ cells were visible in MWA group compared with LQ + MWA group on 12 and 24 hour. More irregularly ablated/unablated interface and islands of active cells were existed in MWA group compared with LQ + MWA group on 12 and 24 hour. Three typical histopathological zones: a typical coagulation necrosis zone, a fibroid tissue zone with infiltration of inflammatory cells, which surrounded the coagulation necrosis zone, and the normal hepatocytes at the outside were existed in MWA and LQ + MWA groups on day 3 and 7. Compared with LQ + MWA group MWA group had thicker fibroid tissue zone,more serious infiltration of inflammatory cells and more irregularly ablated/unablated interface  at 4 time points (Fig. 2).

### Hepatocytes apoptosis

**Figure 3:**
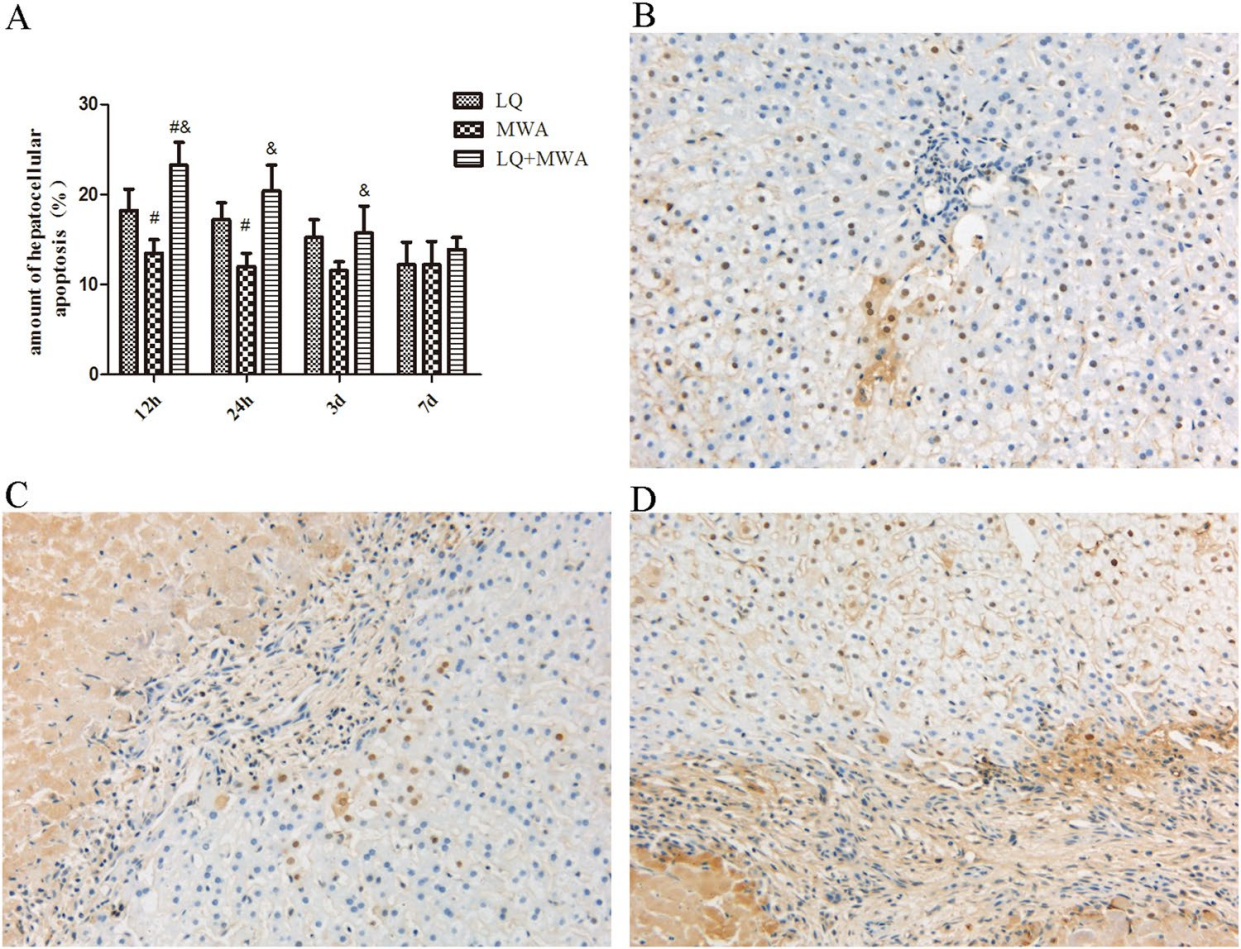
Apoptosis of hepatocytes. The apoptosis was evaluated by TUNEL staining. The amount of apoptotic hepatocytes in different groups and on different time points was shown in (**A**). Representative TUNEL staining was showed in the LQ (**B**), MWA (**C**), and LQ + MWA (**D**) groups on 12 h (magnification ×400). ^#^P < 0.05 VS LQ group; ^&^P < 0.05 VS MWA group.

Apoptosis of hepatocytes was evaluated by TUNEL staining at different time points (Fig. 3). Compared with MWA group(41.52 ± 6.32 cells and 32.73 ± 5.24 cells), large number of apoptotic cells in the LQ group on 12 h and 24 h (51.45 ± 6.05 cells and 48.23c ± 4.53 cells; P < 0.05) and LQ + MWA group on 12 h and 24 h (65.17 ± 6.11 cells and 57.05 ± 7.03 cells; P < 0.001) were noted,The percentage of apoptotic cells in LQ + MWA group was higher than MWA group on hour 12 (P < 0.05) and 24 (P < 0.05), respectively. No significant differences were noted in the 3 groups on day 3 and 7.

### Effect of different treatments on liver function

**Figure 4:**
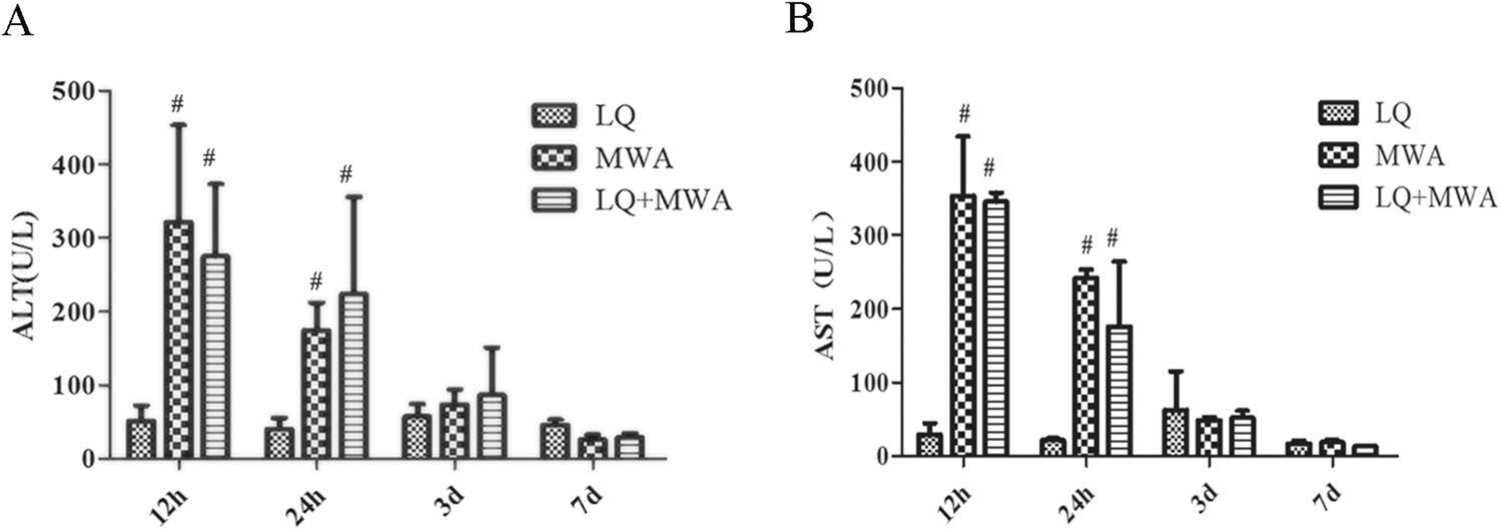
Changes of serum ALT and AST levels. (**A**) Showed the change of ALT level at different time points and in different groups. (**B**) Showed the change of AST level at different time points and in different groups. ^#^P < 0.05 VS LQ group.

Serum ALT and AST were used to evaluate the liver function. We found that there was no significant difference for ALT and AST levels in LQ group at four time points (P > 0.05). ALT and AST levels in MWA and LQ + MWA groups were peaked on 12 hour and dropped on 24 hour and day 3 (P < 0.05). There was no significant difference between MWA group and LQ + MWA on 12 and 24 hour (P > 0.05), however they were significantly greater than LQ group (P < 0.001). On day 3 and 7, no significant differences were identified among the three groups (Fig. 4).

### The effect of different treatments on HSP70 and HIF-1α expression

**Figure 5:**
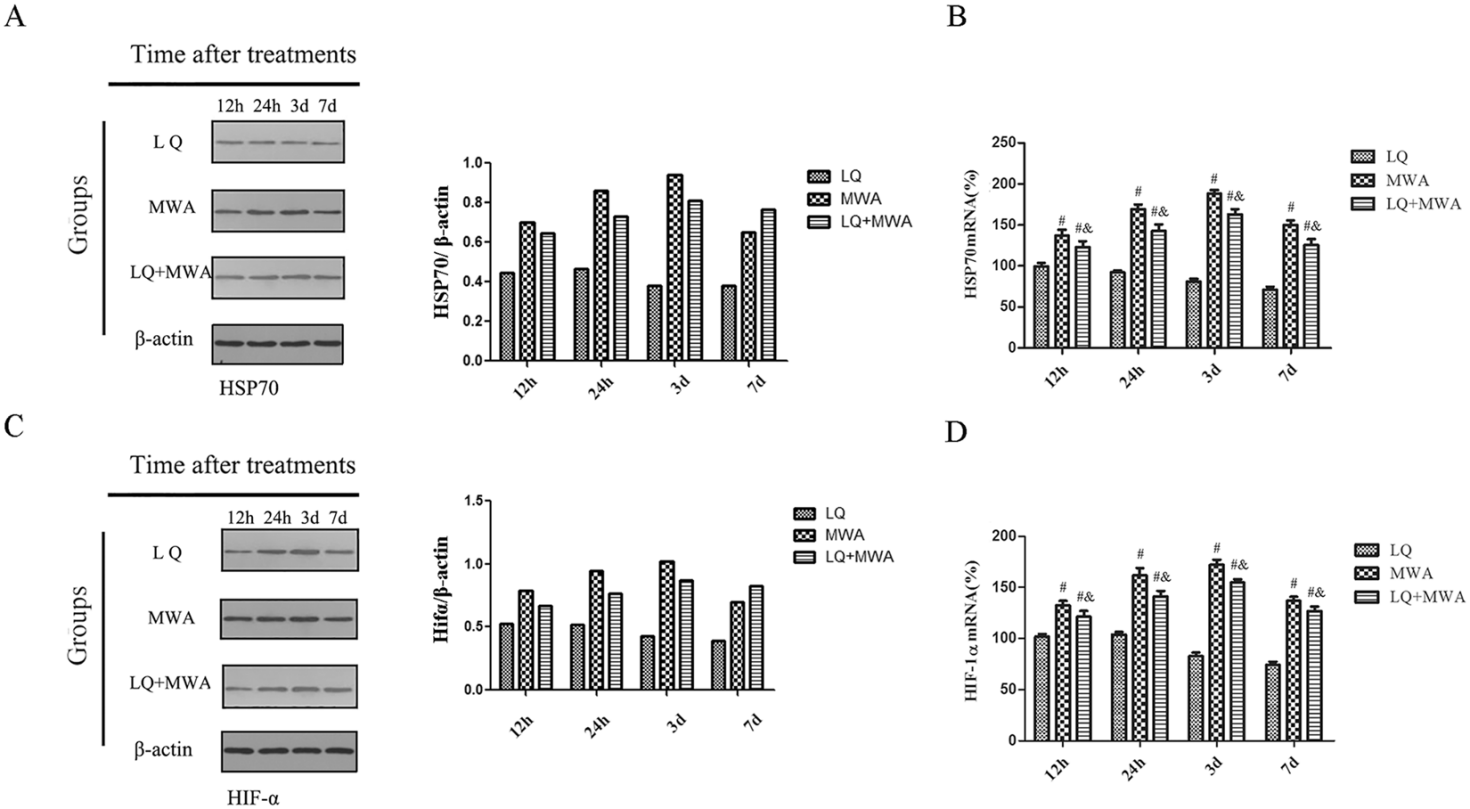
The mRNA and protein expression change of HSP70 and HIF-1α. Western blot and RT-PCR were used to examine the expression of HSP70 and HIF-1α. (**A**) and (**B**) showed the protein expression and mRNA expression of HSP70 in different groups and different time points. (**C**) and (**D**) showed the protein expression and mRNA expression of HIF-1α in different groups and different time points. ^#^P < 0.001 VS LQ group; ^&^P < 0.001 VS MWA group.

**Figure 6:**
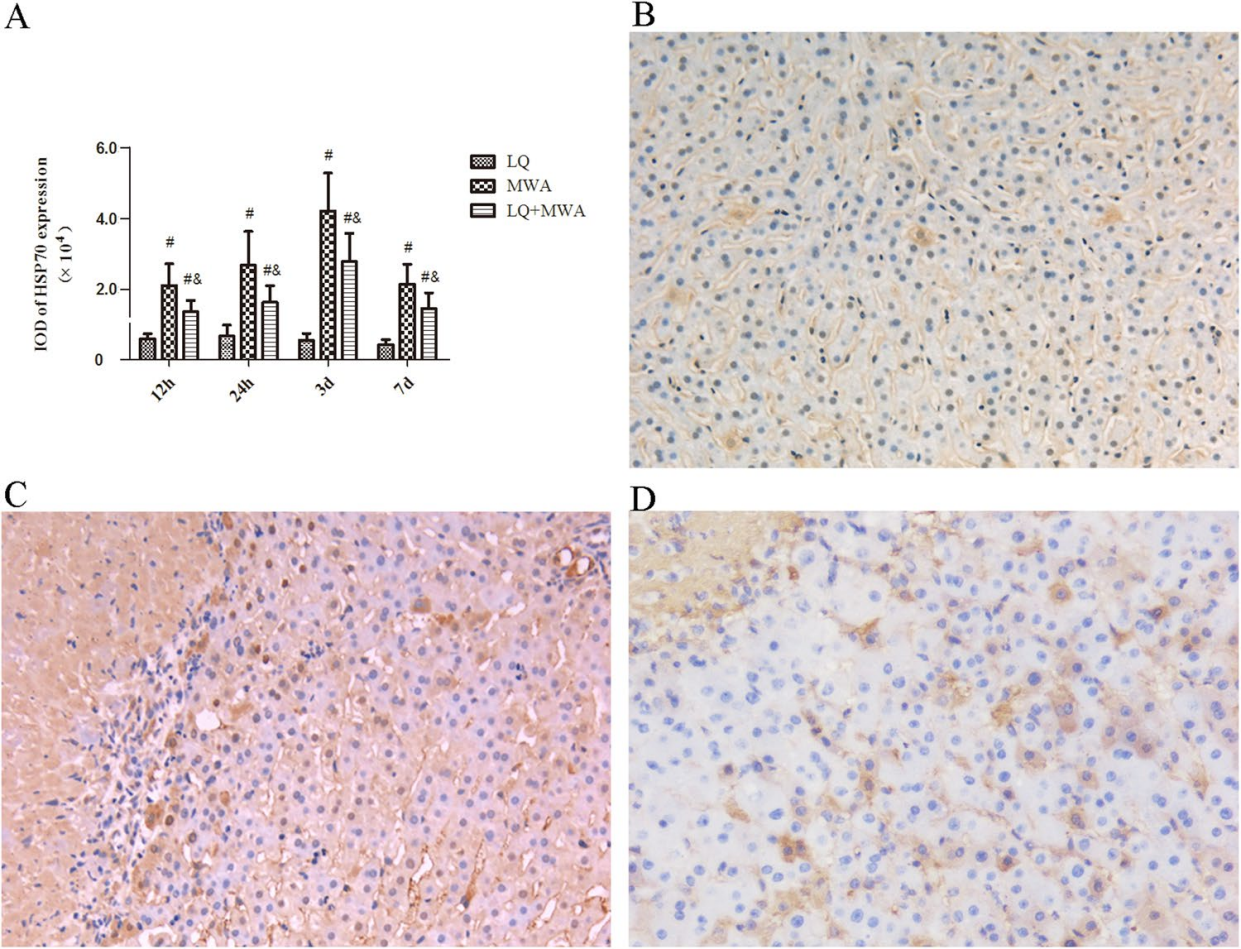
Immunoreactivity of HSP70 in different groups. HSP70 immunoreactivity was measured at the rim of the ablation. (**A**) Showed the IOD of HSP70 expression in different groups on different times. Representative HSP70 expression was showed in the LQ (**B**), MWA(**C**) and LQ + MWA (**D**) groups on day 3 (magnification ×200). ^#^P < 0.001 VS LQ group; ^&^P < 0.001 VS MWA group.

To explore the possible mechanism of LQ, we examined the expression of HSP70 and HIF-1α in liver surrounding ablation tissues. We found that the mRNA and protein of HSP70 were higher in MWA group than LQ group and LQ + MWA group at the all time points (Fig. 5). The IOD of HSP70 was increased on 12 and 24 hour, peaked on day 3, bottomed on day 7 in the MWA and LQ + MWA groups. Compared with LQ + MWA group and LQ group, MWA group had the highest HSP70 on every time point(P < 0.05) (Fig. 6).

**Figure 7:**
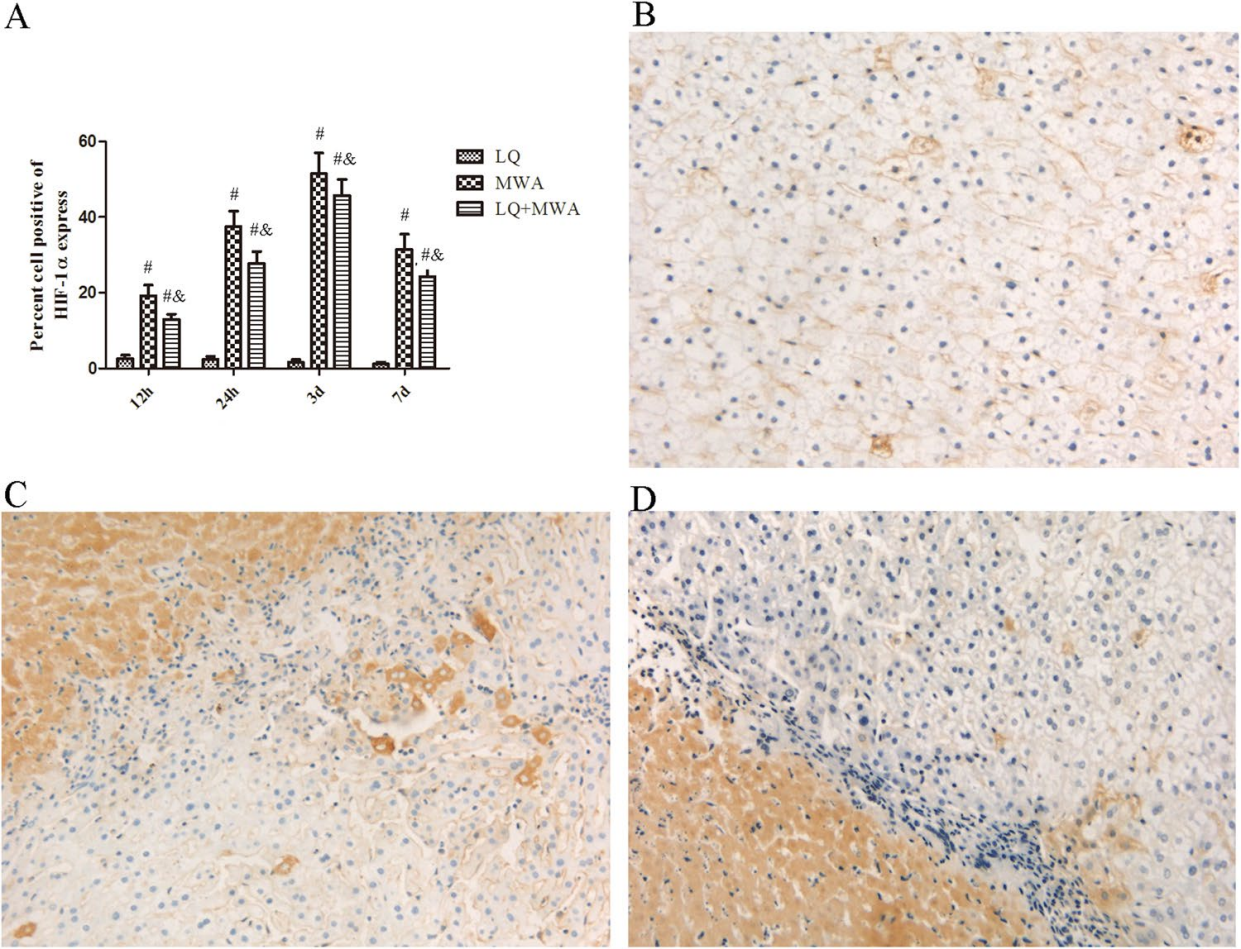
Immunoreactivity of HIF-1α in different groups. HIF-1α immunoreactivity was measured at the rim of the ablation. (**A**) Showed the IOD of HIF-1α expression in different groups on different times. Representative HIF-1α expression was showed in the LQ (**B**), MWA(**C**) and LQ + MWA (**D**) groups on day 3 (magnification ×200). ^#^P < 0.001 VS LQ group; ^&^P < 0.001 VS MWA group.

Western blot and RT-PCR analysis showed that HIF-1α mRNA and protein were both increased in MWA group and LQ + MWA group compared with LQ group at every time point. Compared with MWA group, HIF-1α mRNA and protein expression were lower in LQ + MWA group (P < 0.05). Peak HIF-1α and HIF-1αmRNA expression in the MWA and LQ + MWA groups were also observed on day 3, and dropped on day 7 (Fig. 7).

### The effect of different treatments on TNF-α expression

**Figure 8:**
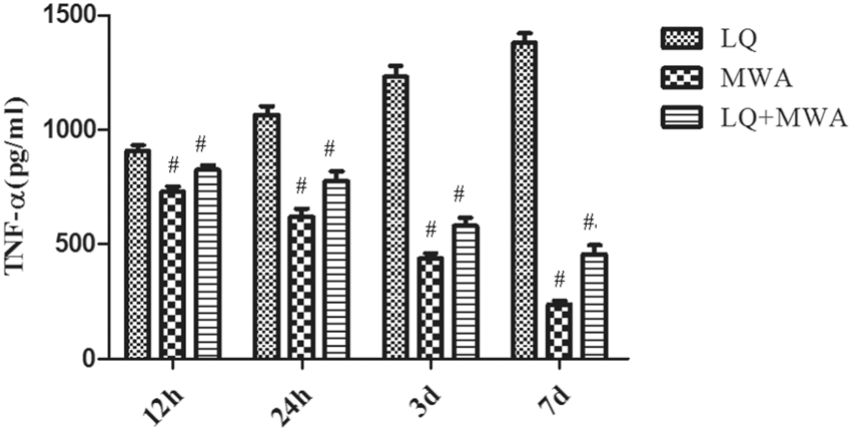
The expression change of TNF-α. The TNF-α expression was detected by ELISA in different groups at different time points. ^#^P < 0.001 VS LQ group.

The level of TNF-α in LQ group was higher than the other two groups at every time point and gradually increased after treatment. There was no statistical difference between MWA group and LQ + MWA group on 12 and 24 hour. The level of TNF-α in MWA group and LQ + MWA group were peaked on 12 and 24 hour, gradually decreased on day 3 and 7 after treatment. There was no statistical difference between MWA group and LQ + MWA group at every time point (Fig. 8).

## Discussion

MWA therapy has become a new therapeutic option for HCC. However, it is difficult in achieving a complete ablation when tumor’s diameter is larger than 3–5 cm^5,9^.

Previous studies showed that exposing to non-lethal hyperthermia resulted in up-regulation of both cellular protective and damaging pathways in the normal or tumor tissues,which produced reversible or irreversible cellular injury^13,14^. Several strategies have been developed to improve the therapeutic efficacy of MWA, including blocking or reducing of tissue blood flow, increasing energy deposition, and modulating tissue characteristics^24^. However, the procedures have been limited to the transcatheter embolization of hepatic arteries or portal veins, complex or controversial operations and surgical Pringle maneuver^5,10,11^. Therefore, less invasive techniques are needed to develop to change the microenvironment of transition region surrounding ablation zone.

Quercetin (3,3′,4′,5,7- pentahydroxy-flavone) is one of the most abundant flavonoids in fruits and vegetables. Large number of studies showed that it has anticancer and anti-inflammatory effects^19^. Liposomal quercetin treatment increased apoptosis and improved RFA-induced tumor destruction by suppression of HSP70 production^14^. Meanwhile, thermal ablation also effectively increased LQ concentrations in tumor tissues to promote apoptosis. These studies imply that LQ has the potential to improve the therapeutic efficacy of MWA. In this study, we found that LQ increased the CV of coagulation necrosis in liver induced by MWA. Regularly ablated/unablated interface was existed in LQ + MWA group. LQ also increased the hepaocyte apopsotis caused by MWA. These results suggest that iposomal quercetin (LQ) can enhance the effect of microwave ablation (MWA) on hepatic parenchyma destruction.

To explore possible mechanism of LQ on hepatic parenchyma destruction induced by MWA, HSP70 and HIF-1α were focused. It is well known that HSP70 has protective effect on cellular injury exposed to hyperthermia^14^. HSP70 is also the primary HSP at periablational rim after focal thermal ablation in normal animal liver and animal tumor models^23^. Some studies showed LQ increased the diameter of the ablation zone by inhibiting HSP70 expression and enhancing apoptosis induced by hyperthermia in the periablational rim at the RFA ablation margin^13,20^. RFA also increased HIF-1α expression in the rim of viable cells immediately adjacent to the ablation zone, which is possibly related to reversible cell injury or regional reduction in tumor perfusion caused by vascular thrombosis^22,25^. So, we examined the expression of HSP70 and HIF-1α.We found that HSP70 induced by MWA was highly expressed at the periablational rim in the rabbit liver, which was in consistent with Duan *et al*. and Nikfarjam *et al*.^26,27^. LQ inhibited the expression of HSP70 induced by MWA. High level HIF-1α was expressed in the marginal area of ablated zone induced by MWA, which may be dependent on secondary effects of hyperthermia (eg, endothelial injury or vascular thrombosis to incite hypoxia^25^. LQ also had the inhibitory effect on HIF-1α expression induced by MWA. These results indicated that LQ suppressed MWA-induced HIF-1α and HSP70.

TNF-α is a critical inflammatory mediator in hepatocyte apoptosis^28^. We also evaluated the influence of LQ on inflammatory response induced by MWA through examining TNF-α level. We found the TNF-α levels in MWA group and LQ + MWA peaked on 12 and 24 hour, and gradually were decreased on day 3 and 7 after treatment. The results suggested that inflammation stimulation and liver injury caused by LQ and MWA were transient and LQ safely increased the ablation effect of MWA. However, the level of TNF-α in LQ group was higher than the other two groups at every time point, suggesting that LQ can induce inflammation itself and the effect last for long time. The contradiction of LQ on inflammation and the possible mechanism need to be further explored in future study.

In conclusion, LQ effectively and safely increase the ablation effect of MWA on liver damage by inhibiting HSP70 and HIF-1α expression. Based on these findings, we will study how LQ improve the effectiveness of tumor control of MWA in rabbit liver tumor model in future studies.
