# Publisher Correction: The influence of liposomal quercetin on liver damage induced by microwave ablation

**DOI:** 10.1038/s41598-017-16208-5

**Published:** 2017-11-17

**Authors:** Xuhua Duan, Pengfei Chen, Xinwei Han, Jianzhuang Ren, Zhaoyang Wang, Guorui Zhao, Hao Li

**Affiliations:** Department of Radiology, The First Affiliated Hospital, Zhengzhou University, No. 1, East Jian She Road, Zhengzhou, 450052 Henan Province People’s Republic of China

*Scientific Reports* **7**:12677; doi:10.1038/s41598-017-13010-1; Article published online 04 October 2017

The original HTML version of this Article contained an error in the title of Figure 5 where the following text was inadvertently inserted:

“comment="Please replace this figure with the attached Figure 5”

This error has now been corrected in the HTML version of the Article; the PDF version was correct from the time of publication.

